# 免疫治疗新浪潮下对中国肺癌免疫临床研究的审思

**DOI:** 10.3779/j.issn.1009-3419.2017.10.06

**Published:** 2017-10-20

**Authors:** 菁菁 柳, 爽 张, 双 李, 颖 程

**Affiliations:** 130012 长春，吉林省肿瘤医院胸部肿瘤内科 Department of Toracic Oncology, Jilin Provincial Cancer Hospital, Changchun 130012, China

**Keywords:** 肺肿瘤, 免疫检查点, 免疫治疗, Lung neoplasms, Checkpoint, Immunotherapy

## Abstract

从抗CTLA-4抗体Ipilimumab在恶性黑色素瘤中获得成功开始，靶向免疫检查点的治疗成为抗肿瘤治疗的有效策略之一，掀起了肿瘤免疫治疗研究的新浪潮。在肺癌领域，国外开展了一系列免疫靶向药物的临床研究，Nivolumab、Pembrolizumab和Atezolizumab相继被批准用于肺癌的治疗，改写了肺癌治疗历史。在中国，也相继开展了肺癌免疫靶向药物的临床研究，本文主要对中国肺癌免疫研究的现状、差距和未来如何创新进行了分析和探讨。

## Abstract (en)

Starting from the success of anti-CTLA-4 antibody in malignant melanoma, targeted immunotherapy has become one of the effective strategies for anti-tumor therapy, and raised a new wave of research on tumor immunotherapy. In the field of lung cancer, a series of clinical studies on immune-targeted drugs have been carried out aboard, nivolumab, pembrolizumab and atezolizumab have been approved for the treatment of lung cancer, which rewrite the history of lung cancer treatment. In China, clinical studies on immune-targeted drugs for lung cancer have also been developed. The present study mainly analyzed and investigated the current situation, gap and future innovation of the research on anti-tumor immunity of lung cancer in China.

免疫系统纷繁复杂，近百年来人们经过漫长的探索，试图通过提高肿瘤细胞的免疫原性和对效应细胞杀伤的敏感性、激发和增强机体抗肿瘤免疫应答、协同机体免疫系统等方法杀伤肿瘤、抑制肿瘤生长^[1]^，但均未取得令人满意的疗效。直至第一个免疫靶向药物抗细胞毒性T淋巴细胞相关蛋白4（cytotoxic T-lymphocyte-associated protein 4, CTLA-4）单抗--Ipilimumab在恶性黑色素瘤中获得成功^[2, 3]^，以及PD-1、PD-L1单抗药物的陆续问世，免疫靶向治疗迅速成为当下肿瘤的研究热点，使肿瘤治疗在继靶向治疗后，迎来了又一次飞跃。而就肺癌领域而言，免疫靶向药物nivolumab、pembrolizumab和atezolizumab相继被美国食品药品监督管理局（Food and Drug Administration, FDA）批准用于非小细胞肺癌（non-small cell lung cancer, NSCLC）的治疗，给肺癌治疗提供了更多的药物选择。在中国，近年来也相继开展了诸多肺癌免疫临床研究，下面就中国肺癌免疫临床研究现状、与国外研究的差距和未来如何创新做一综述。

## 1 国际肺癌免疫临床试验现状

纵观肺癌免疫临床研究浪潮，抗PD-1单抗（nivolumab、pembrolizumab）和抗PD-L1单抗（atezolizumab、durvalumab、avelumab）2大派系、5种药物占领了主要研发市场。梳理这些药物的研发历程，在作用靶点、作用机制、是否需要进行PD-L1检测、治疗线数的排兵布阵和治疗适应症等方面各有千秋。

### 1.1 PD-1单抗

程序性死亡因子-1（programmed death 1, PD-1）是一种Ⅰ型跨膜糖蛋白，属于免疫球蛋白超家族成员，主要表达在活化T细胞上，以单体的形式存在于细胞表面，程序性死亡因子配体（programmed death ligand 1, PD-L1）是PD-1最主要的配体，在免疫应答的负性调控方面发挥着重要作用^[4]^。临床前研究发现，阻断PD-1/PD-L1信号可以促进肿瘤抗原特异性T细胞的增殖，增强免疫反应，从而发挥杀伤肿瘤细胞的作用^[5]^。

作为PD-1单抗的两大代表，nivolumab和pembrolizumab从研究初始即选择了不同的研究人群作为切入点，最早开展临床研究的nivolumab对人群不加选择，同步开展单药治疗和联合治疗研究。CheckMate 063^[6]^和Checkmate017^[7]^研究分别以14.5%和20%的客观缓解率（objective response rate, ORR）和3.0个月的总生存（overall survival, OS）获益超越化疗，为治疗手段匮乏的肺鳞癌提供了新的二线治疗选择，但并没有发现PD-L1表达与药物疗效的相关性。随后非鳞NSCLC的checkmate057^[8]^研究也显示，与化疗比较，nivolumab有2.6个月的OS和19%的ORR获益，并发现治疗疗效与PD-L1的表达相关，被批准用于NSCLC二线治疗，开启了肺癌免疫靶向治疗时代。此外，CheckMate032^[9, 10]^研究也显示出nivolumab单药治疗和nivolumab与ipilimumab联合治疗在复发性小细胞肺癌（small cell lung cancer, SCLC）中分别获得了11%和25%的缓解率，17%和30%的2年生存率，美国国立综合癌症网络（National Comprehensive Cancer Network, NCCN）也据此推荐nivolumab联合Ipilimumab用于复发SCLC的治疗，nivolumab也成为目前唯一在NSCLC和SCLC中均得到推荐的免疫靶向药物。基于联合治疗的较好结果，研究者在CheckMate032研究中增加了一项随机组，2017年美国临床肿瘤学会（American Society of Clinical Oncology, ASCO）年会公布的扩展组第一次报道中显示，242例患者以3:2随机分到nivolumab组或nivolumab 1+ipilimumab 3组，初期的治疗反应与非随机研究类似，联合组和单药组的ORR分别为21%和12%^[11]^。而作为第二个进入临床研发的抗PD-1单抗pembrolizumab，虽然起步略晚，但采取了更加精准的研发策略——研究初始即精确锁定PD-L1阳性人群，Keynote001^[12]^和Keynote 010研究^[13]^率先证实pembrolizumab二线治疗PD-L1≥1%的NSCLC较化疗有显著优势，并确定PD-L1≥50%的患者获益更佳，明确根据PD-L1表达选择获益人群的重要价值，Dako 22C3也成为第一个获批伴随诊断的PD-L1抗体。

在成功获批用于二线治疗后，PD-1单抗又将目光聚焦在一线治疗。在一线治疗中，nivolumab和pembrolizumab分别选择了PD-L1≥5%和≥50%的优势人群。Nivolumab在checkmate026的单药研究中无进展生存期（progression free survival, PFS）为4.2个月，未能超越化疗的5.9个月。而pembrolizumab在一线治疗的Keynote024研究中取得了阳性结果，美国FDA根据17%的ORR提高和4.3个月的OS优势批准pembrolizumab的一线适应证^[14]^。该研究进一步随访结果显示，相比化疗，pembrolizumab有更长的PFS（10.3个月*vs* 6.0个月）和OS（尚未达到*vs* 14.5个月），虽然化疗组有更多患者（80/97）二线接受pembrolizumab治疗，但pembrolizumab组的二线治疗PFS仍然优于化疗组（尚未达到*vs* 8.6个月），这也是第一项在二线治疗选择不均衡的情况下，二线治疗PFS和OS仍然显著获益的研究^[15]^。虽然nivolumab单药在一线治疗中未能取得突破，但采取免疫联合治疗策略的checkmate012研究则让人们看到了nivolumab联合化疗、联合ipilimumab在一线治疗的优势^[16]^，因此nivolumab联合治疗冲击一线是目前的研究热点，正在开展相关的临床研究。而pembrolizumab联合化疗的2期研究--KEYNOTE-021 G队列中^[17]^，联合组的ORR较化疗组提高了26%（55% *vs* 29%），PFS延长了4.1个月（13个月*vs* 8.9个月），这也是首次使NSCLC一线治疗的PFS达到13个月的临床研究，基于此，美国FDA批准pembrolizumab联合化疗用于非鳞NSCLC的一线治疗，这也必将改变NSCLC的治疗格局。

除了晚期患者的治疗外，免疫靶向药物也将目光放到了新辅助和辅助治疗，nivolumab新辅助治疗22例早期、可切除NSCLC（Ⅰb期-Ⅲa期）的Ⅱ期研究^[18]^中，在术前4周给予2个疗程的nivolumab治疗，术后给予标准治疗，研究发现其耐受性良好，无患者出现手术延迟，9/21（43%）患者有显著的病理缓解。这项研究肯定了新辅助免疫治疗的安全性，并观察到一定的疗效，给未来继续开展相关临床研究指明了方向。而新辅助和辅助治疗的系列临床研究也在进行中（Checkmate816、ANVIL、TOP1501、KEYNOTE091等），研究结果值得期待。

### 1.2 PD-L1单抗

PD-L1是PD-1的主要配体，在造血细胞、非造血细胞和肿瘤细胞等多种细胞表面都广泛表达，PD-L1单抗的作用机制是特异性与PD-L1结合，阻断其与PD-1的相互作用，进而抑制PD-1活化，提高T细胞活性。

Atezolizumab作为第一个PD-L1单抗，其研发策略与nivolumab类似，始终将PD-L1阴性患者纳入目标人群，POPLAR研究初步显示atezolizumab较化疗的OS优势^[19]^，与PD-1单抗不同，atezolizumab采用由罗氏诊断研发的抗体SP142，分别评估肿瘤细胞（tumor cells, TCs）和肿瘤浸润免疫细胞（tumor infiltrating immune cell, ICs）上PD-L1的表达水平，并发现PD-L1高表达患者atezolizumab较化疗的OS可延长7.7个月。3期OAK研究进一步验证了POPLAR研究的结果，atezolizumab比多西他赛有更好的生存获益，OS延长了4.2个月，并且在PD-L1表达亚组TC1/2/3或IC1/2/3（PD-L1表达≥1% TC或IC）患者的OS也有显著差异，延长了5.4个月。PD-L1表达越高疗效越好，高PD-L1表达（TC3或IC3）的患者生存获益较多西他赛组改善59%^[20]^。2016年atezolizumab获批成为首个可用于NSCLC二线治疗的PD-L1单抗。基于nivolumab和pembrolizumab的研究经验，atezolizumab也迅速开展单药和联合治疗的一线研究和辅助/新辅助研究，试图寻找突破点。

Durvalumab作为第二个临床开发的PD-L1单抗，结合了其他各药物研究模式的长处，虽然最初研究对象为非选择人群，但很快确定PD-L1高表达人群（≥25%），在联合治疗、一线/辅助治疗领域的研究均已开展，此外与CTLA-4单抗tremelimumab的双免疫联合策略也是重点。Avelumab作为PD-L1单抗的又一新药，研发策略与其他药物相似，将目标人群锁定为PD-L1阳性的患者，并开始初步探索免疫靶向药物联合靶向药物治疗模式的可行性。由此可见，目前国际肺癌免疫靶向药物和相关临床研究众多，研发策略至关重要。

## 2 中国肺癌免疫临床研究现状

### 2.1 国际药物在中国的研究现状

我国作为全球肺癌的高发地区，一直占据了庞大的肺癌新药研发市场。虽然我国尚无获批的免疫靶向药物，但近5年来，国际免疫靶向药物已经在中国开展了10余项临床研究（表 1）。从2013年ipilimumab从中国先后启动了两项肺癌国际多中心3期研究（CA184-156、CA184-104）开始，掀起了中国免疫靶向药物研究热潮。2015年，PD-1抑制剂nivolumab对比多西他赛治疗既往接受过治疗的晚期或转移性NSCLC的checkmate078研究在中国招募患者，这也是首个检测中国肺癌患者PD-L1表达并根据表达情况分层的临床研究。2016年，atezolizumab的IMpower210研究正式启动，pembrolizumab也连续启动Keynote042、Keynote-033、Keynote032三项临床研究，durvalumab+tremelimumab多线治疗实体瘤的Ⅰ期研究也紧随其后进入中国。从这些研究的方案设计也同样可以看出近年来国际肺癌免疫靶向药物研究的走向，即研究对象逐渐由非选择人群逐渐转变为PD-L1阳性患者，研究策略逐渐由单药治疗转变为联合治疗。2017年，中国肺癌免疫靶向药物研究更是出现“井喷”现象，预计至少有10项研究启动。这些研究的共同特点为：以一线治疗为主；单药研究入组PD-L1阳性或高表达患者；免疫联合研究虽入组非选择人群，但要根据PD-L1进行分层，突显出PD-L1检测在免疫靶向治疗中的重要性。

**1:** 国际免疫靶向药物在中国开展的肺癌研究 Study of international checkpoint inhibitors for lung cancer in China

Category	Drug	ClinicalTrials.gov Identifier	Phase	Patients	Status
CTLA-4	Ipilimumab	CA184-156	3	ES-SCLC	Complete
		CA184-104	3	Relapsed NSCLC	Complete
		CA184-153	3	Relapsed squamous NSCLC	Complete
PD-1	Nivolumab	CheckMate 078	3	Relapsed NSCLC	Ongoing
		CheckMate331	3	Relapsed SCLC	Ongoing
PD-1	Perbrolizumab	KEYNOTE042	3	PD-L1+NSCLC	Complete
		KEYNOTE033	3	Relapsed NSCLC	Ongoing
		KEYNOTE407	3	Squamous NSCLC	Ongoing
PD-L1	Atezolizumab	YO29232 (IMpower210)	3	NSCLC	Ongoing
		YO29233	1	NSCLC	Ongoing
PD-L1/CTLA-4	Durvalumab±Tremelizumab	PEARL	3	Untreated NSCLC (PD-L1^*^ high expression)	Ongoing
		NEPTUNE (Durva+Treme)	3	Untreated NSCLC^*^	Ongoing
		D419AC00006	1	solid tumors	Ongoing
CTLA-4: cytotoxic T-lymphocyte-associated protein 4; PD-1: programmed death 1; PD-L1: programmed cell death-1; ES-SCLC: extensive stage small cell lung cancer; SCLC: small cell lung cancer; NSCLC: non-small cell lung cancer.					

总体来说，与国际肺癌免疫靶向研究历程相比，虽然中国研究在启动时间上稍显落后，但随着国外企业对中国市场的重视程度的增加，中国在国际新药研究中扮演了越来越重要的角色，研究模式已经与国际趋于同步。

### 2.2 国内免疫靶向药物研发现状

随着国际免疫治疗新药在中国恶性肿瘤研究领域的全面布局，中国本土医药企业也纷纷开始原研新药的研发。如表 2所示，目前中国有十余家企业已经开始了免疫靶向新药的研发，其中共有4项进入临床研究的PD-1单抗，分别是JS001、SHR-1210、BGB-A317和IBI-308。对于竞争激烈的现状，各药分别采取不同的研发策略，SHR-1210、BGB-A317和JS001率先完成了Ⅰ期研究，2017年ASCO年会，SHR-1210也公布了实体瘤的Ⅰ期研究结果^[21]^，ORR为31.0%，疾病控制率（disease control rate, DCR）为46.5%，3例肺癌中有1例应答。其中BGB-A317在2016年ASCO年会公布了澳洲患者Ⅰ期实体瘤研究结果^[22]^，2017年ASCO又发表了BGB-A317联合聚腺苷二磷酸核糖聚合酶[poly(ADP-ribose)polymerase, PARP]抑制剂BGB-290治疗实体瘤的剂量探索性研究^[23]^，虽然仅入组1例肺癌患者，但也为开展肺癌研究提供了有价值的安全性数据。JS001^[24]^也在2017年公布了Ⅰ期实体瘤研究结果初步证实了该药治疗实体瘤的安全性。就研发速度而言，SHR-1210更胜一筹，在迅速完成Ⅰ期临床研究之后，2017年已经开展单药治疗NSCLC的Ⅱ期临床研究（SHR1210-Ⅱ-201）和联合化疗的Ⅲ期研究（SHR-1210-Ⅲ-303）。除单药治疗外，治疗策略也更多样化，免疫靶向+靶向治疗的Ⅱ期研究即将启动。IBI-308也已经完成了单药以及IO+化疗的Ⅰ期研究，2017年即将启动Ⅲ期研究。除了研究速度加快和研究模式的创新外，更便捷的给药方式也是突破点之一，KN035采用超高浓度制剂、皮下注射的给药方式，便利患者使用，增加依从性，在2016年12月获得国家食品药品监督管理局（China Food and Drug Administration, CFDA）药物临床试验批件，标志着我国首个PD-L1单克隆抗体成功进入临床开发。尽管国内企业陆续在PD-1/PD-L1领域开展研究，但考虑到临床试验存在巨大风险，尤其在国外药物已经上市的背景下，准确定位国产原研新药的治疗策略非常重要。

**2:** 中国肺癌免疫靶向药物研发纵览 Independent research and development of checkpoint inhibitors in China

Category	Drug	Study progress	Approved document time
PD-1	SHR-1210	Phase Ⅰb and phase Ⅱ study are ongoing	2016.2
	BGB-A317	Phase Ⅰ study is ongoing	2016.9
	JS001	Phase Ⅰ study is ongoing	2016.1
	IBI308	Phase Ⅰ study is completed	2016.9
	GLS-010	Clinical trial application stage	2016.4
PD-L1	KN035	Clinical trial application stage	2016.4
	WBP3155	Clinical trial application stage	2016.10
	JS003	-	-
CTLA-4	KN044	-	-
BTLA-4	JS004	-	-
PD-1: programmed death 1; PD-L1: programmed death-1; CTLA-4: cytotoxic T-lymphocyte-associated protein 4; BTLA-4: B-and T-lymphocyte attenuator-4.			

### 2.3 中国肺癌免疫研究与国外的差距

虽然中国已相继开展了一系列肺癌免疫靶向研究，但相比之下，在研药物种类、临床研究数量、治疗模式、研究结果发表数目、获批情况等方面仍与国际存在巨大差距。比如，我国2013年才开始第一项国际药物研究，2015年底第1个国产PD-1单抗JS001才获批进入临床研究，在研药物种类和临床试验数量少，仍停留在跟随阶段，而且由于缺乏中国肺癌患者免疫靶向临床试验数据，CFDA尚未批准任何一种免疫靶向药物，我们对中国肺癌患者免疫治疗的疗效、安全性、优势人群等都缺乏深入的了解。

除了药物之外，另一个更重要的差距在于国际研究采用的PD-L1检测试剂均未进入中国市场，PD-L1检测的可及性非常差。目前虽然少数国内试剂公司推出了PD-L1检测试剂盒，但检测的准确性仍有待验证，加之仍无中国人群PD-L1的cutoff值研究数据，因此临床广泛开展PD-L1检测仍无法实现。而目前国际报道的其他可能与免疫靶向治疗相关的生物标志物，如肿瘤突变负荷（tumor mutation burden, TMB）/新抗原负荷；T细胞受体；肿瘤浸润的淋巴细胞；髓样抑制细胞（myeloid-derived suppressor cells, MDSCs）等标志物在中国人群的研究更是少之又少。此外，中国人群与高加索人群存在显著的基因组学差异，研究^[25]^报道中国人群的表皮生长因子受体（epidermal growth factor receptor, *EGFR*）突变率明显高于西方人群，可达50%左右。有研究^[26]^显示*EGFR*突变患者的TMB低于野生型患者，*TP53*突变及*KRAS*突变的肺癌具有显著增高的TMB。*Meta*分析结果也显示对于*EGFR*野生型患者二线PD-1/PD-L1抑制剂更好，而*EGFR*突变型患者二线化疗更好^[27]^，因此目前绝大多数的免疫研究均排除了驱动基因突变的患者。对于*EGFR*敏感突变率较高的中国肺癌患者，免疫靶向治疗是否可行？目前在研临床研究的研究模式是否适合？如何制定治疗策略等问题仍需要进一步探索。

## 3 中国肺癌免疫研究如何创新

在免疫靶向药物研发风靡全球的大背景下，中国肺癌免疫研究已经落后，若想迎头赶上，除了需要加快免疫药物研发和临床研究的全面布局之外，还需结合中国肺癌人群的特点，在研究切入点、研究策略优化、生物标志物探索等领域开展深层次研究。

### 3.1 尽早开展PD-L1检测

就PD-L1检测而言，作为目前唯一有充分证据与免疫治疗相关的生物标志物，必须在中国尽早落地。目前国际热门PD-1和PD-L1单抗采用的检测平台和试剂各不相同，nivolumab、pembrolizumab和avelumab分别采用Dako28-8、22C3和73-10，atezolizumab和durvalumab分别采用VENTANA SP142和SP263，虽然仍存在如各抗体的亲和力和特异性不同，检测平台和检测阈值差异，肿瘤PD-L1表达时空异质性等问题，但国外已经开展了不同检测平台和抗体的一致性研究，如蓝印计划（The Blueprint Project）、德国一致性研究（German Round Robin）、AZ比较研究（AZ Comparative Study）等。初步结果显示22C3、28-8、SP263具有相似的肿瘤细胞阳性染色百分比，一致性较高，SP142染色阳性的肿瘤细胞较少^[28, 29]^。针对我国尚无任何上述PD-L1检测试剂的窘境，必须加快进口试剂的引进，国产免疫靶向药物临床研究亦应加强与国外试剂公司的合作，加大国内检测试剂的研发力度实现检测的可及性，并尽快开展国产试剂和进口试剂的一致性检验。

此外，目前不同药物所采用的PD-L1 cutoff值尚未统一，在1%-50%不等，而目前中国人群的最佳cutoff值亦不明确。我国正在开展的PD-1抗体SHR-1210二线治疗NSCLC患者的开放性、单臂、多中心的Ⅱ期临床研究中（SHR1210-Ⅱ-20），不但采用国产试剂检测PD-L1的表达，而且会同时验证国产试剂和国外试剂的一致性。在第一阶段按照不同的cutoff值进行分组，从而进一步寻找最佳cutoff值。

### 3.2 探索其他可能的生物标志物

即使免疫靶向药物引领肺癌进入了新的治疗时代，但其有效率也仅在20%左右，PD-L1+的患者也仅在40%上下，仅仅依靠PD-L1一个标志物并不足以帮助我们筛选优势人群，而越来越多的研究结果提示TMB/新抗原负荷；T细胞受体；肿瘤浸润的淋巴细胞；MDSC、巨噬细胞、Treg；干扰素-γ（interferon-γ, IFNγ）/细胞因子/趋化因子；组织相容性复合体（major histocompatibility complex, MHC）Ⅰ/Ⅱ；微卫星高度不稳定（high microsatellite instability, MSI-H）/错配修复缺陷（mismatch repair deficiency, dMMR）；体内细菌微生物等等标志物均与免疫治疗息息相关^[30]^。

MSI-H和dMMR肿瘤可影响细胞内正常的DNA修复，在结直肠癌、子宫内膜癌与胃肠道癌症中最常见，在基于149例MSI-H或dMMR实体瘤的患者的试验中，pembrolizumab治疗的ORR达39.6%，其中78%的患者缓解期达6个月以上。2017年5月，美国FDA快速批准pembrolizumab用于带有MSI-H或dMMR的不可切除或转移性实体瘤的儿童和成年患者，也是FDA有史以来首次基于常见生物标志物而非肿瘤原发部位批准抗癌疗法，足以看出生物标志物在免疫靶向治疗中的重要价值^[31]^。

多项研究证明TMB与免疫靶向治疗的疗效相关，对接受atezolizumab一、二线及以上治疗研究中（POPLAR研究、BIRCH研究和FIR研究）入组的患者进行TMB的回顾性分析显示，TMB在每个样本中都被量化，再根据百份位75（高）、50（中）来评估疗效。一线和二线+患者的中位TMB相似（9/MB和9.9/MB），TMB越高的NSCLC患者（采用FoundationOne测序），接受atezo治疗的疗效更佳^[32]^。而在Checkmate026研究的回顾性分析中，首次在3期研究中证实了TMB对于免疫靶向治疗的预测价值^[33]^。在541例入组患者中，312（57.7%）例患者有可评估的TMB数据。Nivolumab组的TMB高表达比例低于化疗组（29.7% *vs* 39.0%）。所有人群的两组基线特征、PFS和OS相似，但在TMB高表达人群，nivolumab的PFS（中位PFS：9.7个月*vs* 5.8个月；HR=0.62；95%CI: 0.38-1.00）和ORR（46.8% *vs* 28.3%）均显著优于化疗。OS结果相似，但这可能是由于在这一亚组中，化疗组有65%的患者疾病进展后转入到了nivolumab组。美国正在进行一项Ⅱ期研究即探索nivolumab治疗高突变负荷NSCLC的疗效（NCT03023904），也是第一项以TMB为标志物的前瞻性研究，研究模式值得中国研究借鉴，而国内PD-1单抗GLS-010和杰诺单抗已将TMB纳入其临床试验的检测范围。虽然TMB极有希望成为继PD-L1后又一个免疫靶向治疗的标志物，但其cutoff值尚未统一，分析技术要求高，NGS价格昂贵，临床普及仍有很大难度，此外仍需要更多的临床数据加以验证，未来需要进一步探索。

免疫反应系统错综复杂，单一指标难以准确预测免疫靶向治疗的疗效，基于癌症-免疫交互作用的多因子特性，如要实现肿瘤免疫治疗的真正“精准化”，通过生物信息学等多种方法分析和建立多种生物标志物的综合评价体系是未来必然趋势，有研究人员提出了个体中癌症与免疫系统不同互相作用的框架，在Cancer Immunogram中假定了T细胞活性是人类肿瘤中的最终效应机制，并列出这个框架的7个参数，目标是聚焦生物标志物研究，帮助指导治疗选择，但如何实现仍需探索^[34, 35]^。

### 3.3 中国人群不同免疫治疗模式的探索

针对中国*EGFR*突变人群较多的特点，亟需开展更符合中国人群的临床研究。EGFR通过多种机制影响PD-L1表达，TKI治疗后PD-L1表达会发生改变^[36, 37]^，因此以*EGFR*突变人群接受表皮生长因子受体酪氨酸激酶抑制剂（epidermal growth factor receptor tyrosine kinase inhibitor, EGFR-TKI）耐药后给予免疫靶向治疗能否获益为切入点开展研究有重要意义，如目前在研的checkmate722研究即采用单药、IO联合化疗和IO联合IO三种不同模式探索*EGFR*突变患者免疫靶向治疗的潜在获益人群和最佳治疗策略。

此外，基于靶向药物联合治疗的成功经验，随着免疫研究的开展，人们试图将IO治疗的获益最大化，IO联合治疗成为了最热门的研究方向，目前主要联合策略包括IO+化疗、IO+IO、IO+靶向、IO+放疗等等。基于Keynote-21G的研究结果，FDA已经批准pembrolizumab联合化疗用于非鳞NSCLC的一线治疗，而Checkmate012、GP28328（C-E组）研究等初步显示了联合治疗的疗效^[38]^。中国目前也正在开展国际药物的联合治疗研究，国产药物SHR-1210也计划开展联合阿帕替尼、联合化疗治疗NSCLC的2期研究，IBI-308联合培美曲赛/顺铂的3期研究也即将启动。但需要注意的是，在追求疗效的同时，安全性也不容忽视，IO+靶向的TATTON和CAURAL研究已经因为间质性肺毒性的发生率过高终止研究，而目前越来越多的免疫相关不良事件也相继报出，所以联合治疗的安全性仍需谨慎评估^[39]^。此外，联合治疗的优势人群、最佳拍档、给药时机、预测标志物、性价比等问题也需要进一步探索。

## 4 小结

在全球免疫靶向药物研究的浪潮中，中国肺癌免疫治疗道路仍充满诸多挑战，中国需加快研究步伐，搭建顶尖研究框架，探索免疫靶向治疗的生物标志物、耐药机制，开展多中心临床研究，探讨不同治疗模式在中国人群的可行性，提供中国人群研究数据，而政府也应加大对临床研发的支持和药物的审批，鼓励国内企业药物研发，吸引外企药物进入中国，实现免疫治疗的可行性和普及性，才能尽快在肺癌精准免疫治疗领域中占有一席之地。
